# Insight into transketolase of *Pyropia haitanensis* under desiccation stress based on integrative analysis of omics and transformation

**DOI:** 10.1186/s12870-019-2076-4

**Published:** 2019-11-06

**Authors:** Jianzhi Shi, Wenlei Wang, Yinghui Lin, Kai Xu, Yan Xu, Dehua Ji, Changsheng Chen, Chaotian Xie

**Affiliations:** 1 Fisheries College, Jimei University, Xiamen, 361021 China; 2 Key Laboratory of Healthy Mariculture for the East China Sea, Ministry of Agriculture, Xiamen, 361021 China

**Keywords:** *Pyropia haitanensis*, Desiccation tolerance, Integrative omics analysis, Transketolase, Transgenic experiment

## Abstract

**Background:**

*Pyropia haitanensis*, distributes in the intertidal zone, can tolerate water losses exceeding 90%. However, the mechanisms enabling *P. haitanensis* to survive harsh conditions remain uncharacterized. To elucidate the mechanism underlying *P. haitanensis* desiccation tolerance, we completed an integrated analysis of its transcriptome and proteome as well as transgenic *Chlamydomonas reinhardtii* carrying a *P. haitanensis* gene.

**Results:**

*P. haitanensis* rapidly adjusted its physiological activities to compensate for water losses up to 60%, after which, photosynthesis, antioxidant systems, chaperones, and cytoskeleton were activated to response to severe desiccation stress. The integrative analysis suggested that transketolase (TKL) was affected by all desiccation treatments. Transgenic *C. reinhardtii* cells overexpressed *PhTKL* grew better than the wild-type cells in response to osmotic stress.

**Conclusion:**

*P. haitanensis* quickly establishes acclimatory homeostasis regarding its transcriptome and proteome to ensure its thalli can recover after being rehydrated. Additionally, *PhTKL* is vital for *P. haitanensis* desiccation tolerance. The present data may provide new insights for the breeding of algae and plants exhibiting enhanced desiccation tolerance.

## Background

*Pyropia* species are commercially valuable marine red algae that have been cultivated for several centuries in East Asia, including in Japan, South Korea and China [1]. The annual production of *Pyropia* species (many of which were formerly *Porphyra* species) is currently valued at nearly $950 million, with the highest commercial value per unit mass (almost $523 per wet metric ton) among all aquaculture seaweed species [2]. These species are widely used as sources of food, fertilizer, medicine, and chemicals [1]. Kellogg and Lila (2013) reported that *Pyropia* species extracts inhibit the generation of oxygen radicals in vitro systems, suggesting that if they are incorporated into the diet, they may have beneficial effects on many oxidative-damage related conditions (e.g. obesity and cardiovascular disease) [3]. *Pyropia* species may also contribute to the production of “blue carbon” and represent a potentially useful source of biofuel. The expansion of artificial seeding and the development of the floating culture method have considerably enhanced the farming and processing of *Pyropia* species, which represents one of the largest aquaculture industries. *Pyropia haitanensis*, originally identified in Fujian Province in China, is a typical warm temperate zone species, and it accounts for 75% of the total production of *Pyropia* species in China [4].

**Fig. 1:**
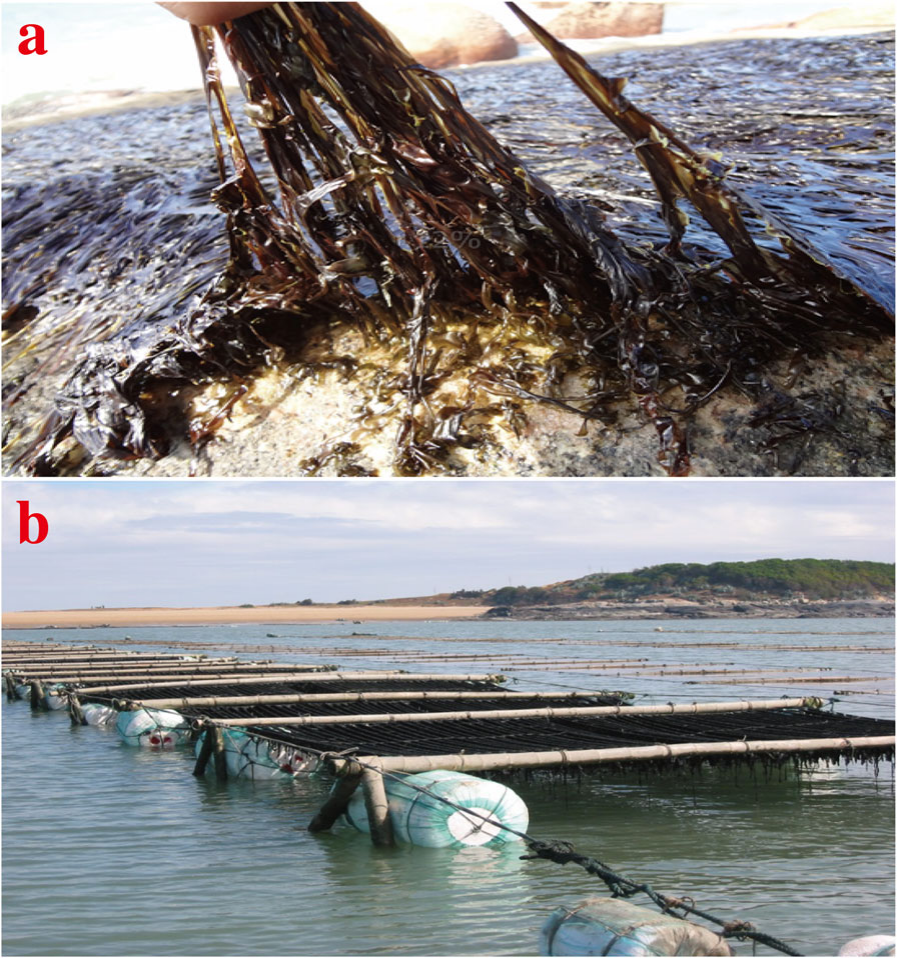
Wild *Pyropia haitanensis* exposed to severe desiccation stress conditions at low tide (**a**). Fishermen regularly use buoys to lift *P. haitanensis* nets above the sea water level to ensure thalli lose water to eliminate wild algae and pathogenic bacteria (**b**)

*Pyropia* species grow in the intertidal zone, where they encounter fluctuations in several abiotic factors due to the tidal cycle. At low tide, *P. haitanensis* is exposed to air, resulting in 80–95% water loss [1]. *Pyropia* species have evolved diverse strategies and mechanisms to withstand such desiccation stress conditions. Consequently, artificially increasing the drying time has been widely used to eliminate wild algae and pathogenic bacteria over the past 50 years, thereby improving the production of *Pyropia* species (Fig. 1). The underlying strategies that enable intertidal macroalgae to survive harsh conditions have not been fully characterized. Studies on the mechanisms of desiccation tolerance have mainly focused on the role of reactive oxygen species (ROS) and changes to the photosynthetic rate. In *P. haitanensis*, antioxidant enzymes accumulate to scavenge ROS produced in response to desiccation conditions [5]. Investigations of many intertidal macroalgae have revealed that decreasing photosynthesis efficiency is another way to reduce ROS production [6]. Additionally, the accumulation of osmotic solutes is an important part of the response to desiccation stress. For example, the floridoside concentration in *P. haitanensis* reportedly increases to protect cells from damages caused by dehydration [7]. However, the protective mechanisms that allow *Pyropia* to adapt to desiccation conditions have yet to be fully elucidated.

Recent advances in “omics” technologies have enabled quantitative analyses of the abundance of biological molecules in a high-throughput manner, making it possible to elucidate the mechanisms underlying the desiccation tolerance of intertidal seaweeds [8]. Because of a lack of genomic information, transcriptomic and proteomic analyses have recently been widely applied to the study of *Pyropia* species [9–11]. Wang et al. (2015) analyzed the transcriptome of *P. haitanensis* exposed to desiccation stress, and identified more than 1500 differentially expressed unigenes (DEGs). Additionally, several metabolic pathways were determined to be involved in the desiccation response, such as trehalose biosynthesis, apoptosis induction, and porphyrin and chlorophyll metabolism [12]. In a study of *Pyropia orbicularis*, 129 proteins were affected by desiccation stress and 56 of these proteins were identified following a bioinformatics analysis. The identified proteins were functionally classified as related to energy and biomolecule metabolism, antioxidant and defense function, and others [13]. Xu et al. (2016) identified 100 differentially expressed proteins (DEPs) in *P. haitanensis* under desiccation conditions, which were mainly associated with photosynthesis and energy metabolism [14]. However, these two investigations of the desiccation tolerance of *P. orbicularis* and *P. haitanensis* were conducted based on two-dimensional electrophoresis (2-DE). The disadvantages of 2-DE include the relatively limited coverage and the potential for inaccurate protein quantification. To overcome some of the limitations of gel-based techniques, isobaric tags for relative and absolute quantitation (iTRAQ) has been recently applied to study *Pyropia* species, thereby improving the throughput and increasing the sensitivity of proteomic studies [15].

The responses of *Pyropia* species to desiccation conditions are affected not only by desiccation-responsive genes or proteins, but also by interactions among the genes and proteins. The relationship between the transcriptome and proteome has not been characterized in *P. haitanensis* exposed to desiccation stress. Therefore, integrating transcriptomic and proteomic approaches in biological studies is warranted because of the generation of complementary data, which enables a more comprehensive molecular characterization. However, gene functions are normally predicted based on BLAST searches. Although previous studies have examined transformed *Pyropia* species, they involved transiently transformed and unstable algae [16, 17]. *Chlamydomonas reinhardtii* was recently described as a good vector for the functional verification of *Pyropia* species genes [16, 17]. Therefore, in the present study, we used *C. reinhardtii* to comprehensively investigate *P. haitanensis* desiccation tolerance. The main objective of this study was to identify the important genes/proteins or pathways through which this intertidal algal species adapts to desiccation stress.

## Results

### Principal component analysis of *Pyropia haitanensis* transcriptomic and proteomic data

**Fig. 2:**
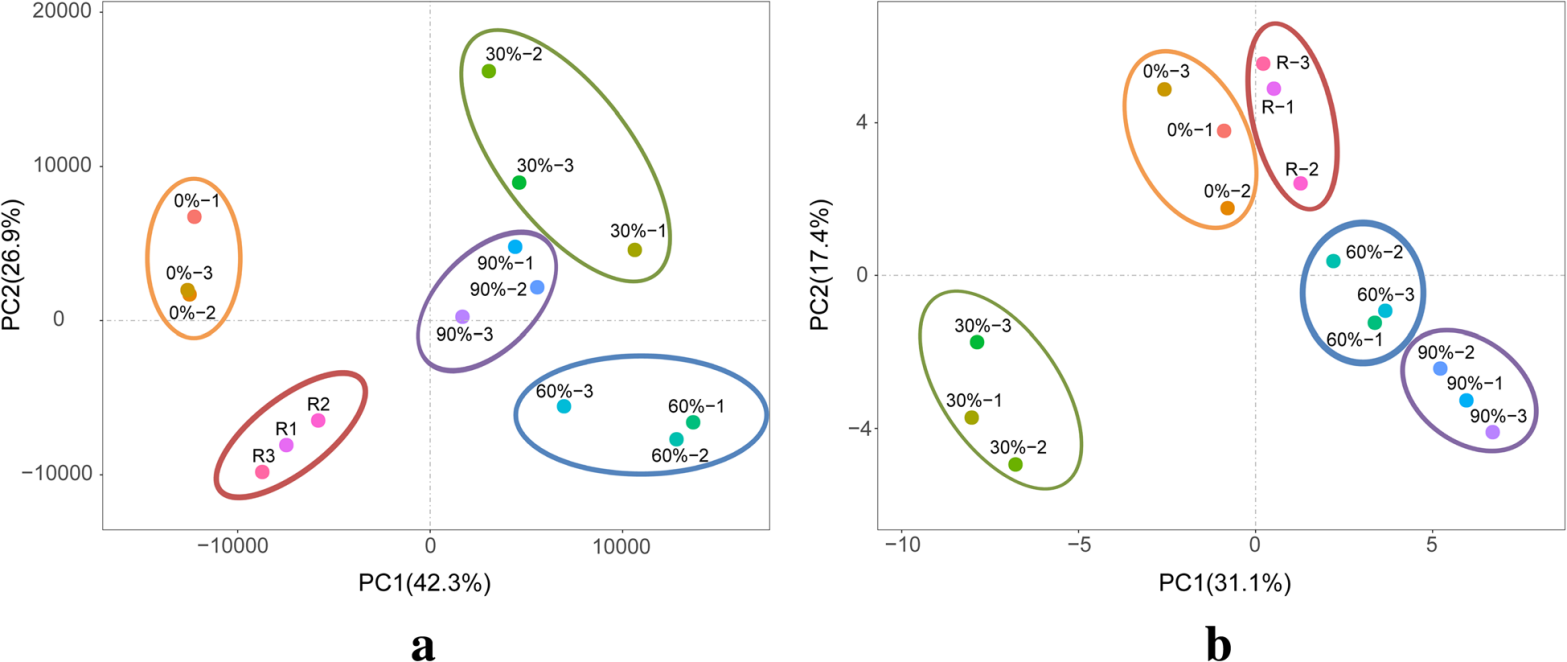
Principal component analysis of *Pyropia haitanensis* transcriptome (**a**) and proteome (**b**) data under desiccation stress. Circles with the same color represent the biological replicates for each treatment. 0%: control; 30%: 30% water loss rate; 60%: 60% water loss rate; 90%: 90% water loss rate; R: rehydration for 2 h

Details regarding the de novo transcriptome assembly and functional annotations are provided in Additional file 1: Table S3. To assess the correlations among the transcriptomic data, a principal component analysis (PCA) was performed for three replicates per treatment (Fig. 2a). The transcriptomic data were similar for the three replicates per treatment. Moreover, different treatments produced distinct results, suggesting that gene expression levels changed during a prolonged exposure to desiccation stress. Additionally, the iTRAQ-based proteomic analysis of all the *P. haitanensis* samples resulted in 149,413 spectra. After eliminating low-scoring spectra, 30,726 unique spectra that met the strict confidence criteria for identifications were matched to 2676 unique proteins. The PCA of the proteomic data produced results that were similar to those of the transcriptomic data (Fig. 2b).

### Expression profiles of differentially expressed genes and proteins

**Fig. 3:**
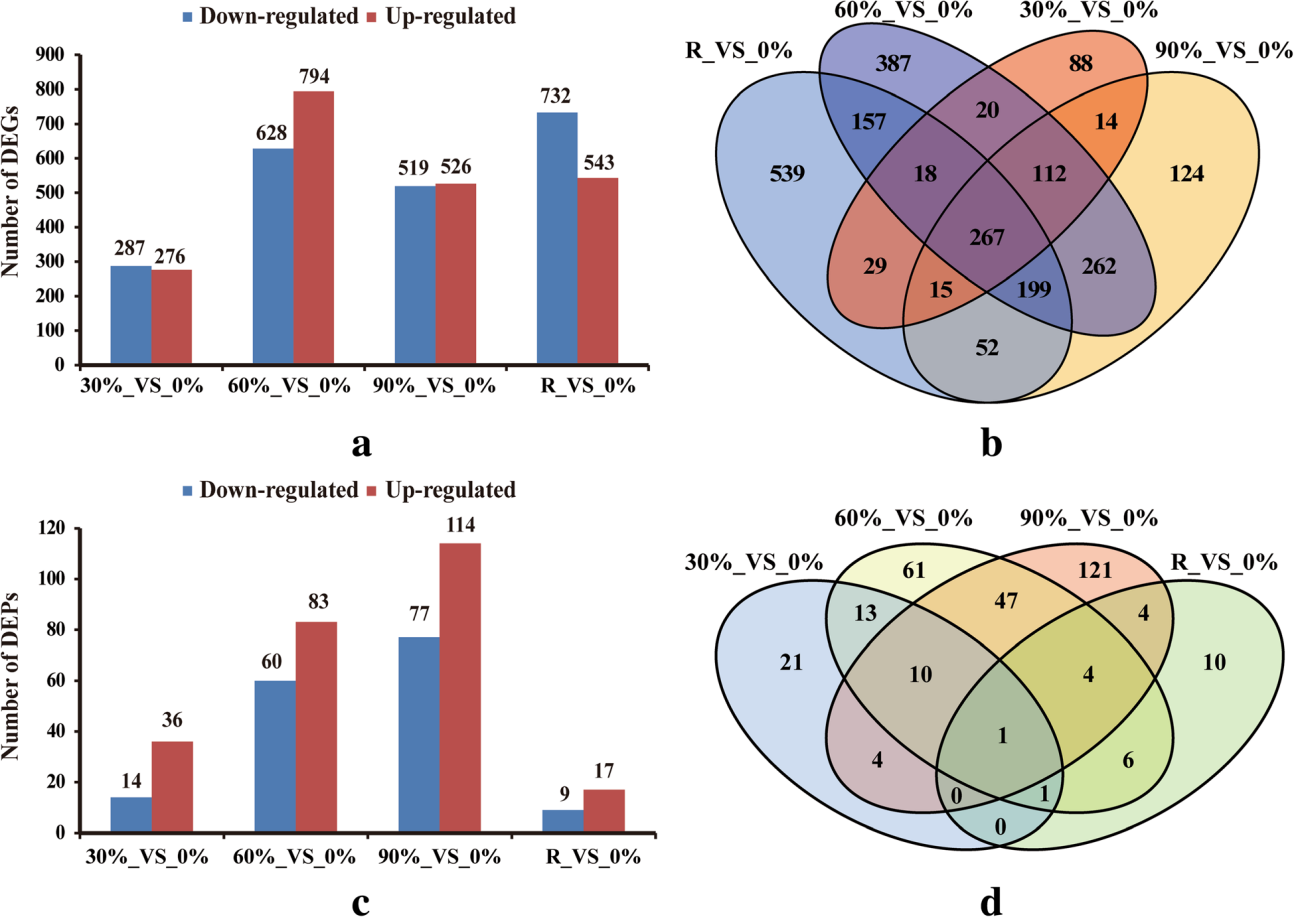
Numbers of differentially expressed genes (DEGs) (**a**) and a Venn diagram of the DEGs (**b**). Number of differentially expressed proteins (DEPs) (**c**) and a Venn diagram of the DEPs (**d**). 0%: control; 30%: 30% water loss rate; 60%: 60% water loss rate; 90%: 90% water loss rate; R: rehydration for 2 h

The unigenes that were up- or down-regulated under desiccation treatments compared to the controls (0%) (log_2_|fold change| ≥ 1 and false discovery rate ≤ 0.001) were determined as DEGs. In response to the 30, 60, and 90% desiccation and rehydration treatments, 276, 794, 526, and 543 unigenes exhibited up-regulated expression, respectively, whereas 287, 628, 519, and 730 unigenes exhibited down-regulated expression, respectively (Fig. 3a). The greatest number of DEGs (1422) was detected in the comparison between the 60 and 0% (control) desiccation treatments. AA Venn diagram analysis of the DEGs under various desiccation stress and rehydration treatments (Fig. 3b) revealed that different genes were responsive to different desiccation conditions, with 267 genes being differentially expressed relative to the control levels in response to all the treatments. A total of 303 DEPs were identified at one or more time-points under desiccation conditions, which represented 11.32% of all the identified proteins. The number of DEPs generally increased along with the desiccation level, but decreased after the 2-h rehydration step. In response to the 30, 60, and 90% desiccation and rehydration treatments, the abundances of 36, 83, 114, and 17 proteins increased, respectively, while the abundances of 14, 60, 77, and 9 proteins decreased, respectively (Fig. 3c). The DEPs were also subjected to a Venn diagram analysis, which revealed that different proteins were responsive to different treatments. Additionally, only one protein was differentially expressed in response to all the treatments (Fig. 3d). Unfortunately, this protein was not functionally annotated.

### Functional analyses of differentially expressed genes and proteins

**Fig. 4:**
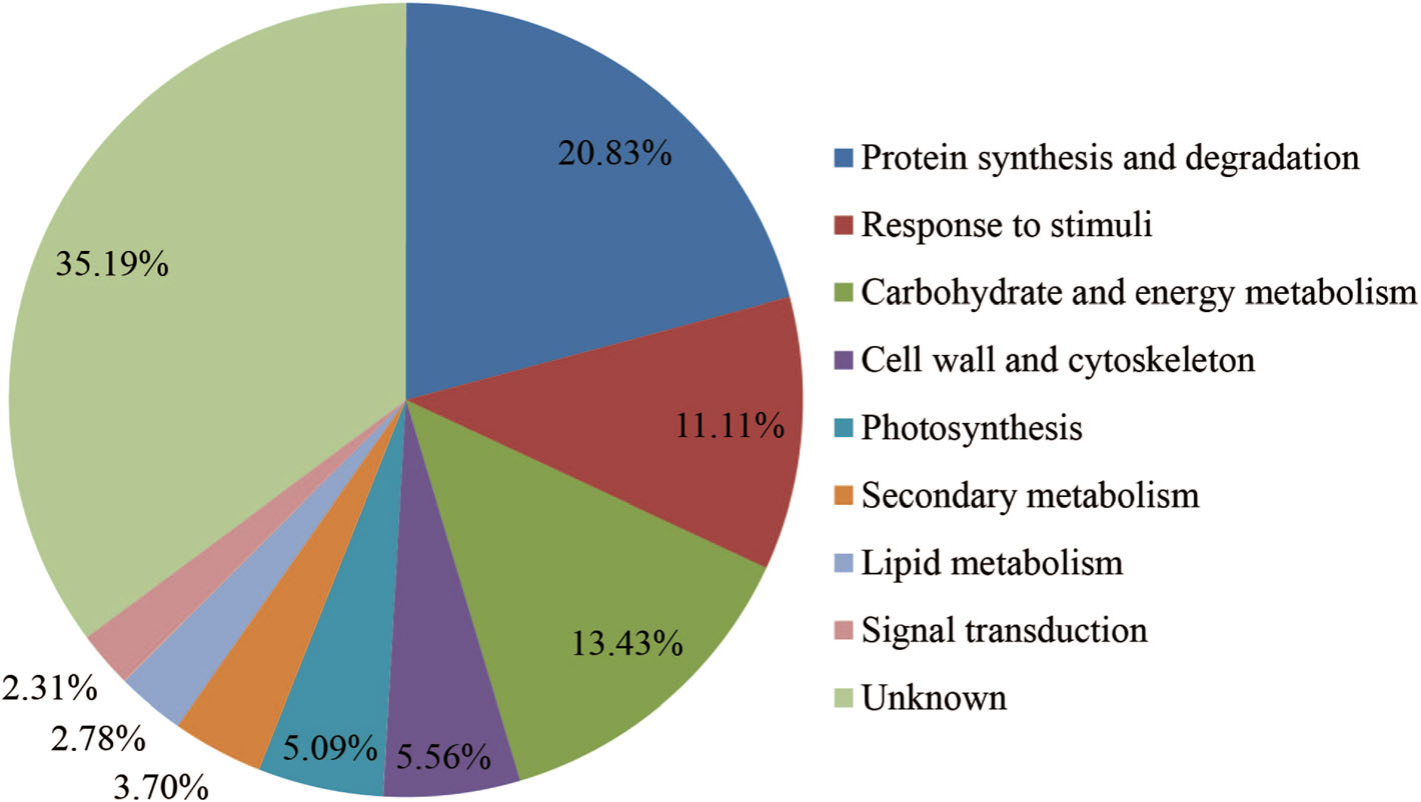
Functional classification of 216 differentially expressed proteins (DEPs) in *Pyropia haitanensis*

**Fig. 5:**
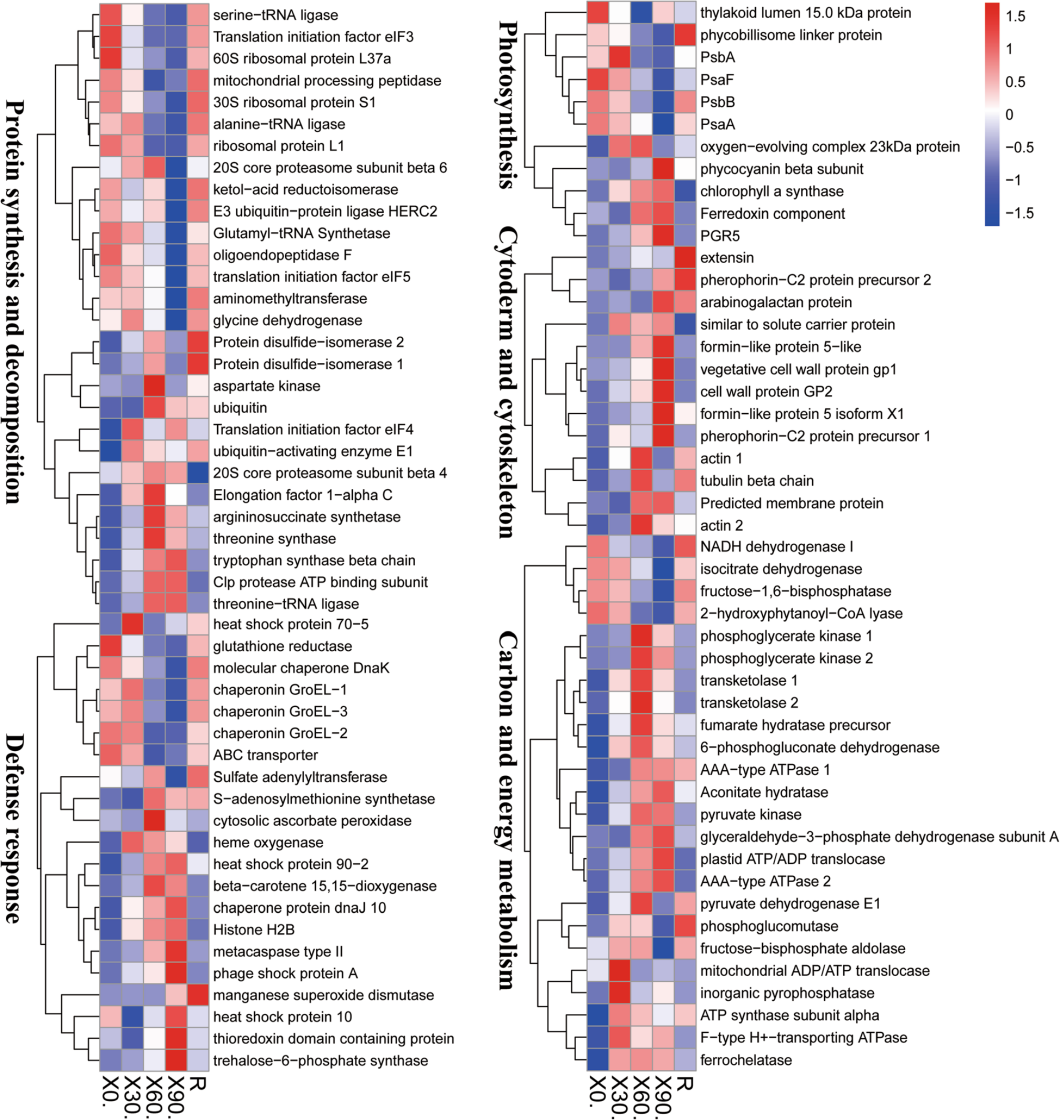
Hierarchical clustering of differentially expressed proteins (DEPs) in *Pyropia haitanensis* with similar functions under desiccation conditions and after rehydration. The protein categories were as follows: protein synthesis and degradation, carbohydrate and energy metabolism, responses to stimuli, cell wall and cytoskeleton, and photosynthesis. 0: control; 30: 30% water loss rate; 60: 60% water loss rate; 90: 90% water loss rate; R: rehydration for 2 h

To obtain a global overview of how *P. haitanensis* responds to desiccation stress, we summarized the changes in gene expression levels according to their putative biological functions. Some important DEGs are listed in Additional file 1: Table S4, including those related to photosynthesis, protein synthesis and degradation, responses to stimuli, the cell wall and cytoskeleton, and energy and carbohydrate metabolism. The functional classification of DEPs (Fig. 4) indicated that most (80.56% of all the DEPs) belonged to one of the following four categories: protein synthesis and degradation (20.83%), carbohydrate and energy metabolism (13.43%), responses to stimuli (11.11%), and hypothetical or unknown (35.19%). The other categories with DEPs were cell wall and cytoskeleton (5.56%), photosynthesis (5.09%), secondary metabolism (3.70%), lipid metabolism (2.78%), and signal transduction (2.31%). A hierarchical cluster analysis grouped the DEPs in response to desiccation stress and rehydration in the following main categories: protein synthesis and degradation, carbohydrate and energy metabolism, responses to stimuli, cell wall and cytoskeleton, and photosynthesis (Fig. 5).

### Verification of differentially expressed genes and proteins

**Fig. 6:**
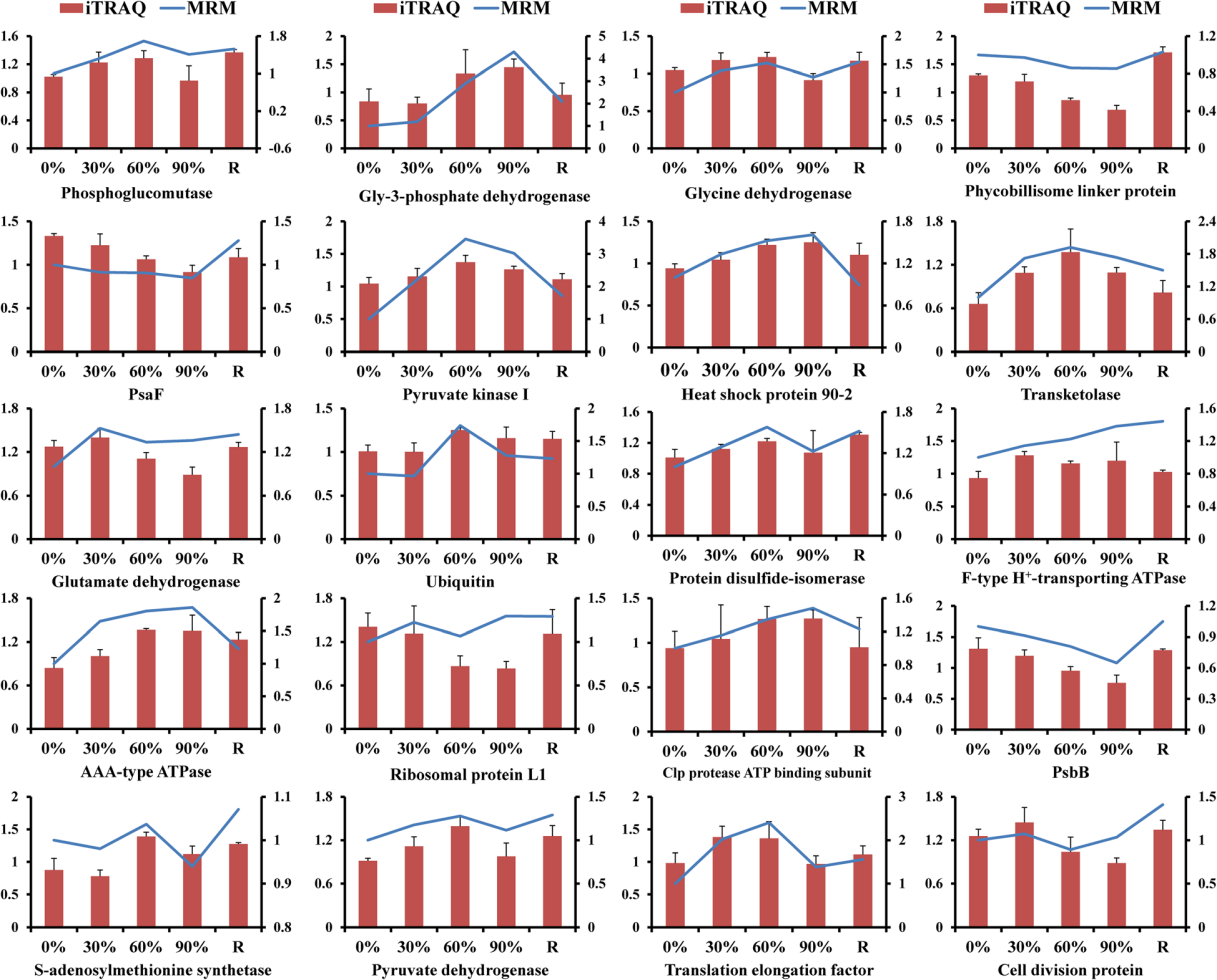
Verification of differentially expressed proteins in *Pyropia haitanensis* under desiccation conditions based on multiple reaction monitoring. 0%: control; 30%: 30% water loss rate; 60%: 60% water loss rate; 90%: 90% water loss rate; R: rehydration for 2 h. The relative changes are shown as log_2_ fold change

The reliability of the RNA-seq data was assessed by a qRT-PCR assay, in which gene-specific primers were designed for 18 randomly selected genes. There was a good correlation between the transcript abundances based on the qRT-PCR assay and the transcript profile revealed by the RNA-seq data (Additional file 1: Figure S1). In total, 30 DEPs were selected to establish an MRM method, which was developed for 20 DEPs. The expression patterns based on the quantitative data from additional MRM analyses were basically correlated with the expression patterns determined by the iTRAQ data (Fig. 6), implying that the iTRAQ results were reliable.

### Integrated analysis of transcriptomic and proteomic data

**Fig. 7:**
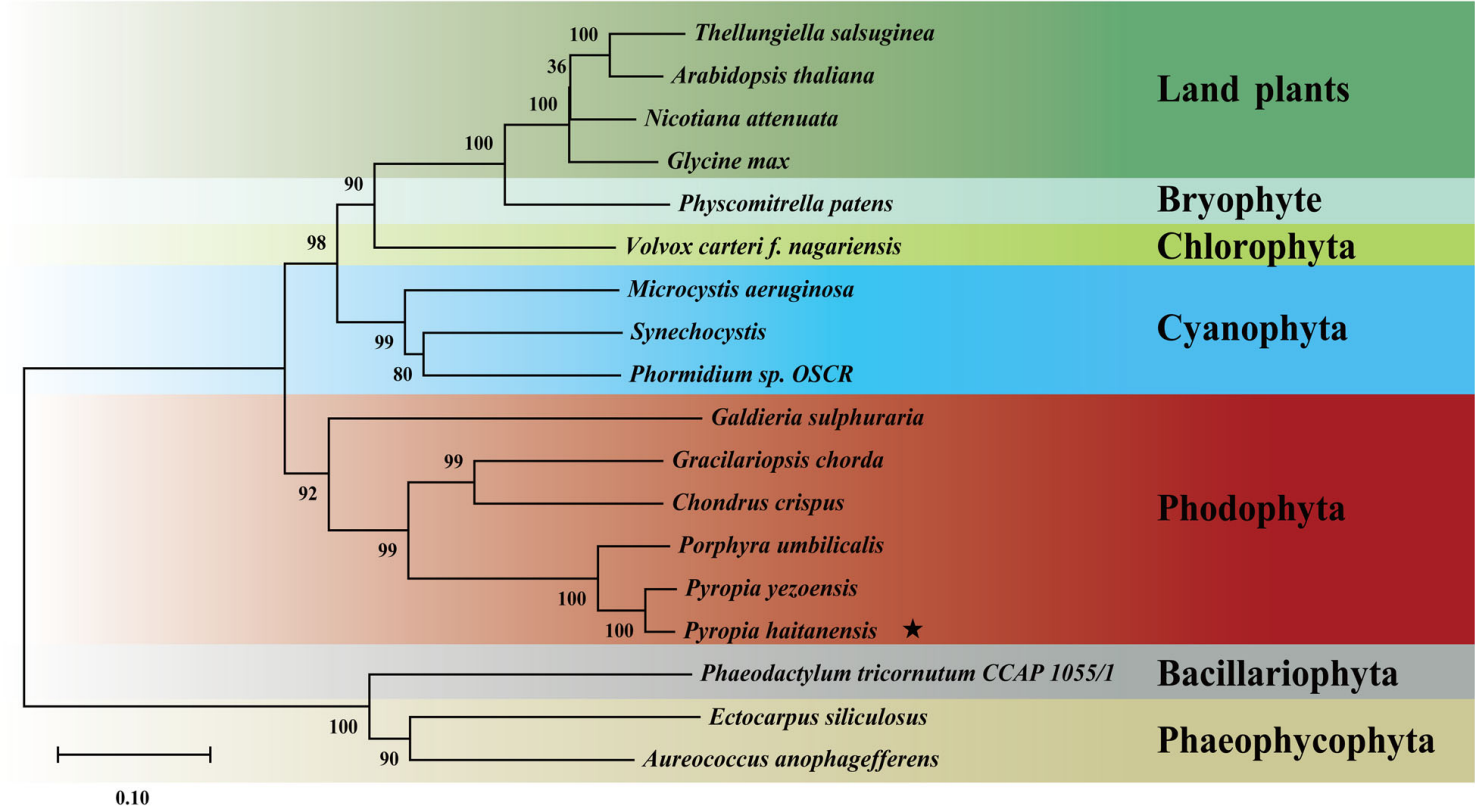
Phylogenetic relationships of transketolase from *Pyropia haitanensis* and representative Cyanophyta, Chlorophyta, Phaeophyta, and land plant species. The phylogenetic tree was constructed using the maximum-likelihood method. Bootstrap values after maximum-likelihood analysis and posterior probabilities after the Bayesian analysis are indicated in the nodes and branches, respectively

The distribution of the corresponding mRNA:protein ratios was analyzed using scatterplots of the log_2_-transformed ratios. Most of the mRNA:protein ratios were concentrated at the center of the plot (quadrant 5), where mRNA and protein levels did not change more than 1.2- and 2-fold, respectively (Additional file 1: Figure S2). Of the 216 identified DEPs, only 48 had corresponding DEGs in the RNA-seq data, which were detected in quadrants 1, 3, 7, and 9. A subsequent analysis was completed with the red scatterplots in quadrants 3 and 7, which indicated that the expression level changes were consistent between the DEGs and corresponding DEPs. A total of 26 DEGs were identified (Table 1). Moreover, the up-regulated DEGs were associated with specific activities (e.g., pentose phosphate pathway, ROS scavenging, inositol metabolism, Calvin cycle, photosynthetic electron transport, glycolysis, and cell wall modification). In contrast, the down-regulated DEGs were related to molecular chaperone, protein processing, and photosynthesis. Both transketolase genes and encoded proteins were upregulated under 30, 60 and 90% desiccation conditions (Table 1). A phylogenetic tree supported the existence of a sister-group relationship between *P. haitanensis* and other Rhodophyta species, but implied that *Pyropia* species diverged from Cyanophyta, Chlorophyta, Phaeophyta, and land plants (Fig. 7).

**Table 1:** Differentially expressed genes that changed in accordance with their homologous differentially expressed proteins

Gene ID	(log_2_ FC)	Proteins (log_2_ FC)	Annotation	Function
30%_VS_0%				
c12879_g1	2.13	0.72	Transketolase	Pentose phosphate pathway
c10632_g1	4.83	0.32	Hypothetical protein	Hypothetical or unknown
c13641_g2	2.09	0.39	Heme oxygenase	ROS scavenging
60%_VS_0%				
c12879_g1	5.40	1.06	Transketolase	Pentose phosphate pathway
c13777_g1	4.50	0.53	Myo-inositol dehydrogenase	Inositol metabolism
c12790_g1	4.28	0.71	Phosphoribulokinase	Calvin cycle
c10632_g1	8.22	0.38	Hypothetical protein	Hypothetical or unknown
c13780_g20	1.46	0.50	Glycosyl transferase	Secondary metabolism
c12732_g3	3.09	0.84	Proton Gradient Regulation 5	Photosynthetic electron transport
c24990_g1	2.43	0.53	Unnamed protein product	Hypothetical or unknown
c10780_g1	1.42	0.72	Ferredoxin	Photosynthetic electron transport
c13180_g3	−2.24	− 0.27	Molecular chaperone DnaK	Molecular chaperone
c11346_g1	−1.00	−0.57	Chaperonin GroEL	Molecular chaperone
c1535_g1	−2.46	−0.51	Unnamed protein product	Hypothetical or unknown
c9789_g1	−1.13	−0.43	Putative protein disulfide-isomerase	Protein processing
c13728_g2	−1.95	−0.86	Hypothetical protein	Hypothetical or unknown
c12496_g2	−3.06	−0.37	Pyrophosphate--fructose 6-phosphate 1-phosphotransferase	Carbon metabolism
90%_VS_0%				
c12879_g1	3.73	0.73	Transketolase	Pentose phosphate pathway
c13133_g2	3.87	0.35	Predicted protein	Hypothetical or unknown
c13777_g1	3.26	0.62	Myo-inositol dehydrogenase	Inositol metabolism
c13015_g1	1.06	0.47	Probable dehydrogenase	Hypothetical or unknown
c11143_g1	3.12	0.79	Glyceraldehyde-3-phosphate dehydrogenase	Glycolysis
c12940_g1	1.21	0.51	Unnamed protein product	Hypothetical or unknown
c14325_g1	1.27	0.39	Ferrochelatase	Secondary metabolism
c13026_g1	1.01	0.62	Unnamed protein product	Hypothetical or unknown
c11214_g2	1.42	0.50	Unnamed protein product	Hypothetical or unknown
c12732_g3	1.64	1.30	Proton Gradient Regulation 5	Photosynthetic electron transport
c6671_g1	3.80	0.66	Vegetative cell wall protein	Cell wall modification
c13180_g3	−1.98	−0.43	Molecular chaperone DnaK	Molecular chaperone
c12337_g1	−1.47	−0.54	Photosystem I subunit III	Photosynthesis
Rehydration_VS_0%				
c10380_g1	4.78	0.37	Pherophorin-C2 protein	Cell wall modification
c12790_g1	1.14	0.55	Phosphoribulokinase	Calvin cycle
c12337_g1	−3.33	−0.29	Photosystem I subunit III	Photosynthesis
c13612_g4	−3.97	−0.44	Uncharacterized protein	Hypothetical or unknown

### *PhTKL* enhances the osmotic tolerance of transgenic *Chlamydomonas reinhardtii*

**Fig. 8:**
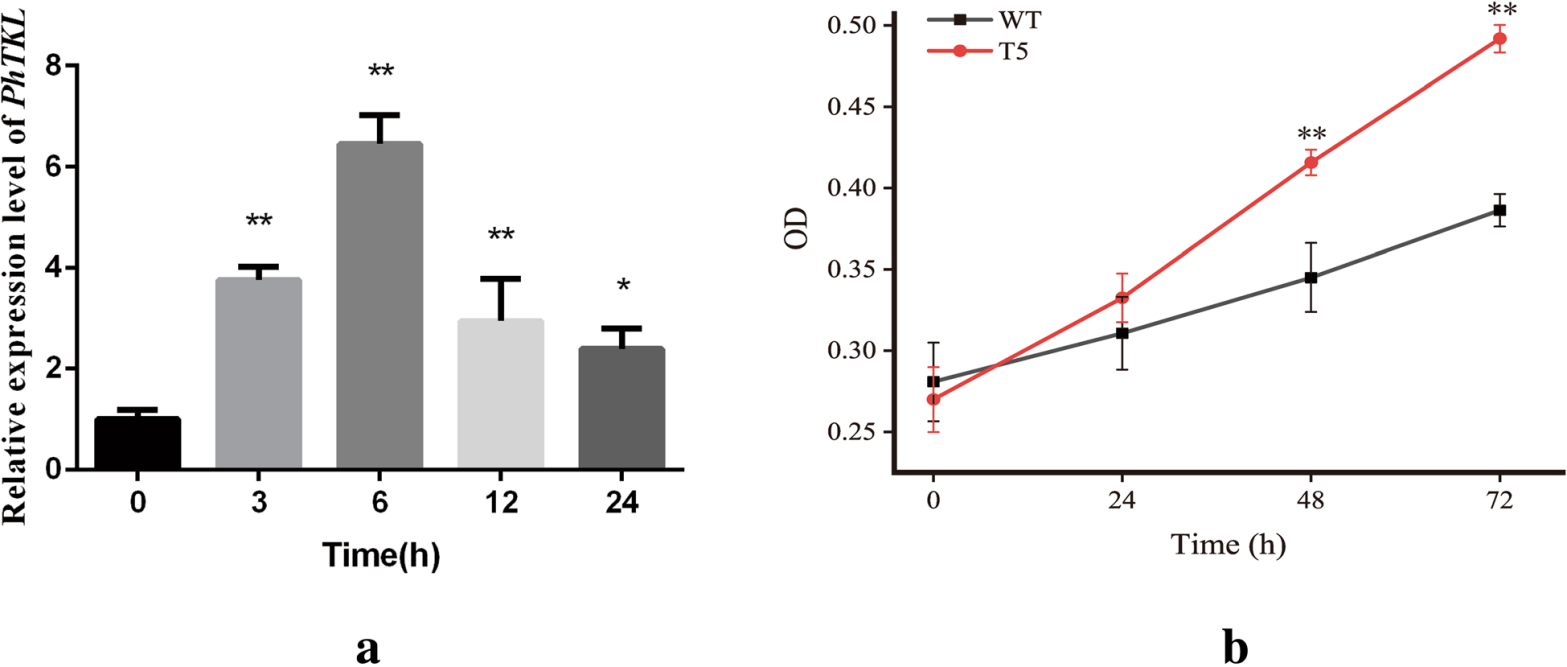
Effects of *PhTKL* on transgenic *Chlamydomonas reinhardtii*. (A) Relative *PhTKL* expression level under osmotic stress conditions; *significant difference (*p* < 0.05), **highly significant difference (*p* < 0.01). (B) Growth of transgenic *C. reinhardtii* (T5) and wild type (WT) cells under osmotic stress conditions

Putative *C. reinhardtii* transformants detected based on their resistance to hygromycin were assessed by PCR with *PhTKL*-specific primers (Additional file 1: Table S2). The expected PCR products were generated for all the randomly selected hygromycin-resistant lines, but not for the wild-type plants, verifying the integration of *PhTKL* into the genome of the hygromycin-resistant lines (Additional file 1: Figure S3). The first transgenic lines (T_1_) underwent transcriptional and morphological analyses to assess their osmotic tolerance. Specifically, a qRT-PCR assay was completed to determine *PhTKL* expression levels induced by osmotic stress. *PhTKL* expression was up-regulated by osmotic stress, with peak levels observed at 6 h after treatments (Fig. 8a). To functionally characterize *PhTKL*, *C. reinhardtii* cells over-expressing *PhTKL* were cultivated in TAP medium containing 200 mM mannitol. The wild-type and transformed *C. reinhardtii* cells initially grew similarly, but the growth rate of the transgenic cells was obviously greater after 2 days (Fig. 8b).

## Discussion

In this study, we found that *P. haitanensis* rapidly regulated its physiological activities in response to desiccation stress before losing 60% of its cellular water. A prolonged exposure to desiccation conditions stimulates a series of protective responses, but *P. haitanensis* may also rapidly establish acclimatory homeostasis in its transcriptome and proteome to ensure recovery after being immersed in seawater. We identified 2283 DEGs and 303 DEPs, but only 26 DEGs exhibited expression-level changes that were consistent with the changes in the abundance of the corresponding DEPs. The poor correlation between transcriptomic and proteomic data was likely due to post-transcriptional regulatory activities under desiccation conditions. These findings emphasize the need to investigate molecular processes at transcriptional and translational levels. Such an integrated analysis unveiled a set of stress-inducible genes/proteins, but also revealed the molecular mechanism associated with *P. haitanensis* desiccation tolerance.

### Photosynthesis

The absorption of light is the initial step of photosynthesis. Phycobilisomes, which consists of phycobiliproteins (PBPs) and linker polypeptides, are the major light-harvesting complexes in *Pyropia* species [15]. On the basis of their spectral properties and pigment compositions, PBPs can be divided into three main groups, namely phycoerythrin, phycocyanin, and allophycocyanin. In addition to the up-regulation of genes related to PBPs, we observed an elevated phycocyanin production in *P. haitanensis* exposed to desiccation stress (Table 1, Fig. 5). This phenomenon was also observed in *P. orbicularis*, which is distributed along the upper rocky intertidal zone of the Chilean coast [13]. An integrated analysis of the transcriptome and proteome revealed down-regulated DEGs or DEPs related to photosynthesis (Table 1). The increased abundance levels of PBPs may capture light energy and further decrease ROS production. In this study, the *PsbA*, *PsbB*, and *PsaA* transcripts and encoded protein levels were maintained under mild desiccation conditions (30% water loss) and decreased under moderate desiccation conditions (60% water loss), but could subsequently recover to their original levels (Fig. 5). Similar results were obtained for an examination of *P. haitanensis* photosynthetic parameters, which indicated that the maximum and effective photochemical quantum yields of photosystems II (PSII) were strongly inhibited when the water loss exceeded 60%; however, they recovered to 79 and 60% of their original levels, respectively, in the absence of desiccation stress [14]. Thus, *P. haitanensis* may have evolved to accommodate periodic dehydration and rehydration cycles.

The physical properties of the photosynthetic apparatus are important for drought-tolerant plants. Photosynthetic components are very sensitive and prone to damage, and they must be maintained or repaired quickly after rehydration. To prevent the accumulation of photodamaged PSII, the repair process includes assembly of the oxygen-evolving machinery [18]. In the present study, a 23 kD oxygen-evolving complex protein was upregulated under moderate desiccation stress (Fig. 5). It has the function to stabilize the Mn_4_CaO_4_ cluster [19]. Desiccation stress usually damages the oxygen-evolving complex of PS II [20, 21]. In contrast, in the current study, its abundance in dehydrated *Pyropia* thalli may help to maintain the structure of oxygen-evolving complex during moderate desiccation conditions, which may influence *Pyropia* species’ responses to desiccation stress. Additionally, the energy transfer to the reaction centers of PS II and PS I lead to electron transfer to ferredoxin and then the reduction of NADP^+^ to NADPH. NADP^+^ is a major acceptor of electrons in PS I [22]. In the current study, ferredoxin was upregulated under severe desiccation stress (Fig. 5), indicating that it might function to promote the reduction of NADP^+^ to NADPH, decrease the transport of electrons from PSI to molecular oxygen and inhibit ROS producion [22]. The accumulation of NADPH in *P. haitanensis* provides electrons for P700+ and drives the cyclic electron flow via PSI under severe desiccation conditions (84% water loss) and recovery during rehydration, while the PS II activity was abolished at a 76% water loss [23]. This indicated that the cyclic electron flow around PSI was still active after the inactivation of the linear electron flow under severe desiccation conditions. Similarly, the PS I-driven cyclic electron flow of the desiccated *P. yezoensis* thalli at a 87% water loss could be rapidly restored upon re-hydration, which was faster than PS II [24]. Furthermore, PROTON GRADIENT REGULATION5 (PGR5) has been suggested to mediate electron transfer via the ferredoxin-mediated cyclic electron flow [25]. PGR5 dependent regulation of electron transfer and proton motive force are significant for the protection of PS I against photodamage [26]. Mature Arabidopsis overexpressing PGR5 survive exposure to high light and desiccation stress better than wild-type [27]. Here, we also found that PGR5 genes and encoded proteins were upregulated during severe desiccation condition (Fig. 5, Table 1). These results suggest that the cyclic electron flow around PS I might play a significant physiological role in response to desiccation and rehydration, and may be an important factor enabling *Pyropia* to adapt to intertidal environments.

### Cell wall and cytoskeleton

Cell wall modifications are important for plant acclimations to environmental stresses [28]. In *Ulva compressa*, cell walls structural changes provide protection from mechanical damage during periodic desiccation–rehydration cycles, thereby enabling this species to adapt to the intertidal zone [29]. Herburger et al. (2019) reported that the accumulation of pectin homogalacturonan in the filament cell wall of Zygnema resulted in increased desiccation tolerance [30]. Under desiccation conditions, *P. haitanensis* cells shrink, which is similar to the effects of desiccation on other algae cells [7]. Our data also revealed the increased abundance of glycoproteins associated with cell wall modifications (i.e., extensin, pherophorin-C2 protein, arabinogalactan protein, and cell wall protein) (Fig. 5). Salt stress induces an increase in expansin levels in *P. haitanensis*, which enhances the retention of water [31]. These changes might increase cell wall flexibility and help *P. haitanensis* adjust to environmental conditions. The production of a membrane protein was also induced by desiccation (Fig. 5). Membrane proteins functioning as receptor-like kinases are believed to perceive changes in the extracellular space and transmit their signals into the cell [32]. The expression levels of many genes encoding receptor-like kinases are induced by abiotic stresses, thereby amplifying the signals for necessary stress-adaptive responses, including the maintenance of cell wall integrity [33]. These results highlight the cell wall changes that enable *P. haitanensis* to adapt to desiccation conditions.

The cytoskeleton is a highly dynamic cell component that mainly comprises microtubules and actin filaments. The cytoskeleton of plant cells can undergo substantial changes when subjected to abiotic stresses [34]. The degradation of cytoskeletal proteins might result in cytoskeletal disassembly as well as cell structure changes in *Physcomitrella patens* under desiccation stress [35]. The actin microfilaments of the green algal species *Klebsormidium crenulatum* are obviously disrupted in response to desiccation, possibly because of changes to the actin microfilament phosphorylation status [36]. The destruction of the cytoskeleton might be a consequence of desiccation stress among higher plants and green algae [37]. However, recent studies have uncovered a close relationship between the cytoskeleton and abiotic stress tolerance in *Pyropia* species [13, 15]. The abundance of three actin and tubulin proteins in *P. haitanensis* increased at a WL rate of 60% (Fig. 5). Actin abundance also increases in the aquatic bryophyte *Fontinalis antipyretica* Hedw under dehydration conditions, and increases in β-tubulin contents may help preserve cell structures in *F. antipyretica* after rehydration [34]. The results of the current study suggest that the cytoskeleton affects the cellular homeostasis of *P. haitanensis* under desiccation conditions. However, the associated mechanisms underlying the desiccation tolerance of *Pyropia* species will need to be characterized in future studies.

### Osmotic adjustment

Trehalose is a non-reducing disaccharide of glucose that functions as a compatible solute that helps stabilize biological structures under abiotic stress [38]. The abundance of trehalose-6-phosphate synthase (TPS), which catalyzes the synthesis of trehalose, began to increase in response to the 60% desiccation treatment (Fig. 5). This result was consistent with the findings that *TPS* expression levels increase in response to considerable water losses [39]. In *Selaginella lepidophylla* under dehydration conditions, trehalose accumulates to 12% of the plant dry weight and protects proteins and membrane structures [40]. Additionally, *TPS* is reportedly over-expressed in *P. orbicularis* under desiccation conditions [41]. The *P. haitanensis* thalli may positively adjust the osmotic potential via the considerable synthesis of trehalose and other osmolytes to adapt to short-term 100‰ hypersaline stress [31]. Thus, trehalose appears to be an important osmoprotectant in *P. haitanensis*.

### Protein synthesis and degradation

Abiotic stress is associated with an enhanced risk of improper protein folding and denaturation [38]. In the current study, desiccation stress decreased the expression levels of genes and abundances of proteins related to protein synthesis, including a ribosomal protein, translation initiation factor, and aminoacyl-tRNA synthetase (Table 1, Fig. 5). High-temperature stress can also repress the synthesis of *P. haitanensis* proteins [15]. These changes may decrease the overall production of proteins, and consequently eliminate some misfolding and denaturation. Nevertheless, misfolded proteins inevitably accumulate in cells under abiotic stress conditions, which necessitate ubiquitination to target proteins for degradation by a proteasome [42]. Our results indicated that the expression levels of genes related to protein degradation decreased during desiccation treatments (Table 1), but the abundance of the corresponding proteins increased (Fig. 5). In an earlier investigation of *Ulva prolifera*, it was observed that the abundance of DEPs may significantly increase or decrease without a change in the corresponding mRNAs levels [43]. Differences between the DEGs and DEPs imply that changes in protein profiles owing to desiccation stress may be controlled at the post-transcriptional level. Moreover, the increased abundances of DEPs linked to ubiquitination may help *P. haitanensis* to remove misfolded proteins and decrease the risk of cell damage. In addition, our data clearly indicated that the abundance of DEPs in this pathway were significantly affected in cells with 60% water loss (Fig. 5), which is similar to the photosynthesis-related DEPs. These observations are further evidence that the 60% WL rate is an important threshold for *P. haitanensis* responses to desiccation.

In addition to ubiquitination, chaperone activities may also control the accumulation of misfolded proteins induced by abiotic stress in *P. haitanensis* [9, 44, 45]. For example, Hsps are common chaperones that maintain functional protein conformations under abiotic stress conditions by preventing nonnative protein aggregations and by refolding proteins into native conformations [46]. Previous 2-DE investigations concluded that Hsps were highly abundant in *Pyropia* exposed to desiccation stress [13, 14]. In contrast to the transcriptome results, Hsp70, Hsp90, DnaJ, and Hsp10 abundances increased (Fig. 5), which highlights the post-transcriptional regulation occurring in *P. haitanensis*. Moreover, decreasing proteins production (Table 1) is an efficient strategy for avoiding protein misfolding and denaturation in *P. haitanensis* [15]. This may explain any decreases in molecular chaperone levels and protein processing activities. The expression patterns of these DEGs imply that *P. haitanensis* blades may limit damages caused by desiccation to repairable levels.

### Response to stimuli

One of the mechanism underlying desiccation-tolerant plants involves the activation of several processes that minimize damage associated with specific by-products (e.g., ROS) [47–49]. In an earlier investigation of *Zygnema circumcarinatum*, the expression levels of genes encoding glutathione-S-transferase, peroxisomal catalase, peptide methionine sulfoxide reductase and peroxiredoxin were higher in Group P2 than Group L, indicating a more severe ROS stress response in *Z. circumcarinatum* filaments grown on agar plates (desiccation stress) than grown in liquid culture [50]. In various intertidal species, desiccation also significantly enhances ROS production, leading to an increase in antioxidant contents, including enzymes [5, 6]. In this study, the abundances of ascorbate peroxidase, heme oxygenase, manganese superoxide dismutase, and thioredoxin increased under high (≥ 60%) desiccation stress conditions (Fig. 5). In *P. haitanensis*, an antioxidant system scavenges for redundant ROS to maintain a certain redox balance during short-term 110‰ hypersaline stress [27]. The expression levels of *PhSOD*, *PhCSD1* and *PhCSD2*, were highly induced by the O_2_·^−^ content in *P. haitanensis* under desiccation and high-temperature stress [51]. Low ROS production and high enzymes activities and antioxidant contents have been revealed during the responses of intertidal seaweed to desiccation stress [6]. Thus, *P. haitanensis* appears to deploy detoxifying enzymes and proteins to control the ROS level, and an antioxidant system likely enhances the survival of blades under desiccation conditions. Furthermore, programmed cell death is crucial for plant development and defense mechanisms [52]. In the current study, we detected an increase in metacaspase abundance (Fig. 6), which is consistent with a previous analysis of *P. haitanensis* responses to high-temperature stress [15]. Consequently, programmed cell death is an important part of *P. haitanensis* defense responses.

### Carbohydrate and energy metabolism

In response to stresses, plants initiate a series of events linked to carbohydrate and energy metabolism [53]. A previous 2-DE study revealed that *P. haitanensis* increases the production of energy-related proteins to withstand desiccation conditions [14]. Our iTRAQ results presented herein confirmed that proteins mediating carbohydrate and energy metabolism are needed for desiccation tolerance, including 29 DEPs related to glycolysis, the tricarboxylic acid (TCA) cycle, and pentose phosphate pathway (Fig. 5). Glycolysis is a central metabolic pathway that provides energy and generates precursors for the synthesis of primary metabolites [54]. Our transcriptome analysis indicated that most DEGs participating in glycolysis exhibited up-regulated expression (Table 1). Similarly, the abundances of all the glycolysis-related DEPs increased following the desiccation treatment (Fig. 5). Respiratory activities proceed along with the mitochondrial reactions of the TCA cycle, ultimately generating large amounts of ATP [55]. The abundances of pyruvate dehydrogenase, aconitate hydratase, and fumarate hydratase, which belong to this pathway, increased under desiccation stress (Fig. 5). Additionally, the pentose phosphate pathway also contributes to the production of energy and sugar phosphates under abiotic stress conditions [56]. Environmental stresses cause *Pyropia* species to increase their glycolytic activities and induce the TCA cycle [13, 15]. These observations suggest that energy metabolism is a complex process, and that *P. haitanensis* blades require considerable energy to tolerate desiccation stress conditions.

**Fig. 9:**
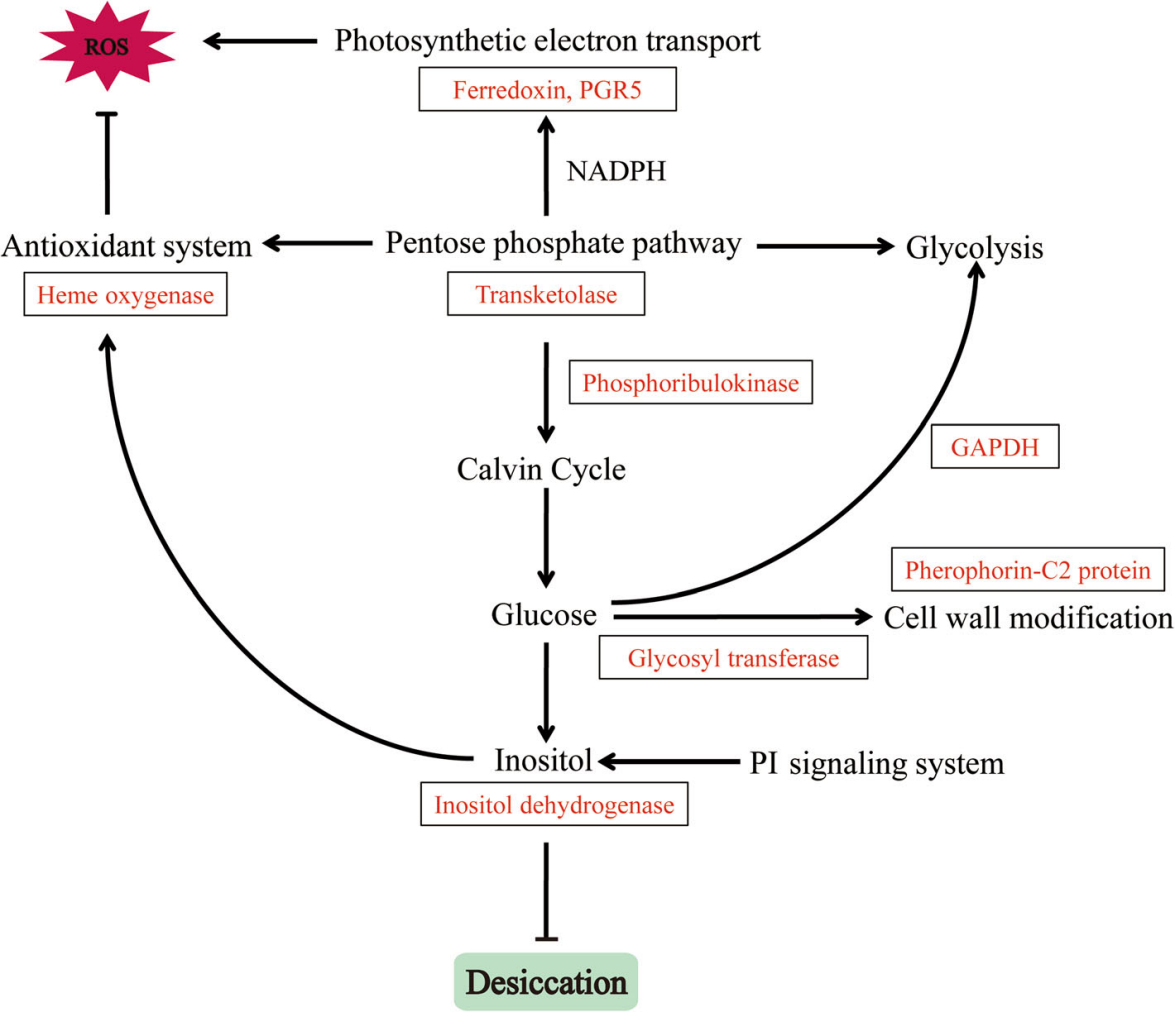
Integrated analysis of transketolases and metabolic pathways in *Pyropia haitanensis* under desiccation conditions

ATKL, which is crucial for the pentose phosphate pathway, was detected in response to all the desiccation treatments (Table 1). TKL generates NADPH and sugar phosphate intermediates [57]. As a multi-functional pathway, the pentose phosphate pathway is closely associated with stress tolerance. In *U. prolifera*, NADPH derived from the pentose phosphate pathway provides electrons for the cyclic electron flow around PSI, which enhances the survival of thalli exposed to salt stress [58]. The observed increases in ferredoxin and PGR5 levels imply a similar role under desiccation conditions. Intermediates of the pentose phosphate pathway, such as fructose 6-phosphate and glyceraldehyde 3-phosphate, are also involved in glycolysis [47]. Our data indicate that glyceraldehyde-3-phosphate dehydrogenase production in *P. haitanensis* was induced by desiccation stress, suggesting that the pentose phosphate pathway regulates the glycolysis pathway. In addition to providing reducing equivalents and energy, the pentose phosphate pathway has been linked to the Calvin cycle via phosphoribulokinase [59]. In this study, phosphoribulokinase production increased under mild and moderate desiccation conditions, implying that the *P. haitanensis* Calvin cycle is maintained for the accumulation of carbohydrates. Glycosyltransferase helps synthesize hemicellulose, pectin, mannans, and various glycoproteins, making it critical for plant cell wall metabolism [60]. Additionally, inositol is a sugar-like carbohydrate produced by most plants, and it regulates cellular water pressure and exhibites antioxidant capacities [61]. Regarding ROS scavenging, heme oxygenase helps protect cells from oxidative damage [62]. On the basis of these results, we speculated that the pentose phosphate pathway indirectly affects the antioxidant activities of *P. haitanensis*. Moreover, Gao et al. (2013) reported that in *P. haitanensis*, the cyclic electron flow rate around PSI increases under desiccation conditions, whereas the linear electron flow around PSII is suppressed. The PSI-driven cyclic electron flow may be more rapidly restored than PSII activities following re-hydration, possibly in part because of the enhanced cyclic electron flow around PSI due to the NADPH derived from the pentose phosphate pathway [23]. We functionally characterized *PhTKL* based on the results of our integrated analysis. We observed that *PhTKL* expression was induced by osmotic stress (Fig. 8a). Moreover, transgenic *C. reinhardtii* over-expressing *PhTKL* grew better than control cells on TAP medium containing a high mannitol concentration (Fig. 8b). Therefore, this is the first report describing the importance of *PhTKL* for desiccation tolerance (Fig. 9). Furthermore, a phylogenetic tree including *PhTKL* suggested that *Pyropia* species diverged from Cyanophyta, Chlorophyta, Phaeophyta, and land plant species (Fig. 7). The specific regulatory mechanism associated with *PhTKL* will need to be characterized in future studies, which may provide new insights into the stress tolerance of *P. haitanensis*.

## Conclusions

We conducted an integrated transcriptome and proteome analysis of *P. haitanensis* exposed to desiccation stress. Our data revealed a clear *P. haitanensis* response to desiccation conditions, with a 60% WL rate representing an important threshold for this response. The *P. haitanensis* thalli were able to grow normally under mild desiccation conditions; however, a WL rate exceeding 60% activated desiccation-tolerance mechanisms that mainly affected photosynthetic activities as well as the cell wall and cytoskeleton, the pentose phosphate pathway, and the antioxidant system. Our integrated analysis of the transcriptome, proteome, and transgenic *C. reinhardtii* implied that the pentose phosphate pathway plays a central role in the desiccation tolerance of *P. haitanensis*. This is the first study to elucidate the transcriptomic and proteomic changes in *P. haitanensis* in response to desiccation stress. The data presented herein provide new insights into the molecular mechanism underlying the desiccation tolerance of macroalgae.

## Methods

### Materials and stress treatment

*Pyropia haitanensis* strain Z-61 was obtained from the Laboratory of Germplasm Improvements and Applications of *Pyropia* at Jimei University, Fujian, China [63]. Blades were cultured in Provasoli’s enriched seawater in a growth chamber set at 21 °C with a 12-h light: 12-h dark photoperiod (50 μmol photons m^− 2^ s^− 1^). Provasoli’s enriched seawater was refreshed every 3 days. The Z-61 blades that grew to 15 ± 2 cm were randomly selected for a stress treatment. Three biological replicates for each sample were analyzed in the subsequent experiments. The following four desiccation levels were set based on water loss: 0% (control), 30% (mild desiccation), 60% (moderate desiccation), and 90% (severe desiccation). The rehydration (R) treatment involved submerging blades for 2 h after the water loss reached 90%. The temperature was held constant for the desiccation and rehydration treatments. The water-loss (WL) rate was calculated with the following published equation with some modifications [5]. First, we designed a pre-experiment to estimate the initial water content (IWC) of Z-61 strain. In total, 12 blades which were 15 ± 2 cm in length were selected and weighed after removing the surface water with gauze (W0). Then, they were dried in an oven at 60 °C to a constant weight (Wi). IWC was calculated using the following formula:

$$ \mathrm{IWC}={\sum}_{i=1}^{n=12}\left(W0- Wi\right)/\left(W0\times 12\right) $$

For the desiccation treatment, we first obtained the weight (W0) after drying the surface of blades with gauze. Then, based on the IWC and specific water loss rate (WL), we calculate the actual weight (Wd) according to the following formula:

$$ \mathrm{WL}=\left(W0- Wd\right)/\left(W0\times IWC\right)\times 100\% $$

Then, the Z-61 blades were exposed to the air and weighed every 15 min. When Wd was reached, the blades were stored in liquid nitrogen for further RNA and protein extraction.

*Chlamydomonas reinhardtii* strain “CC-400 cw15 mt+” was provided by Prof. Tse-Min Lee (Institute of Marine Biology, National Sun Yat-sen University), and used to assay the physiological function of candidate genes. Specifically, *C. reinhardtii* cells were grown in Tris-acetate-phosphate (TAP) medium at 25 °C with shaking at 100 rpm under 14 h:10 h light/dark (L:D) photoperiod with cool fluorescent light (50 μmol photons m^− 2^ s^− 1^). For the osmotic stress treatment, cells were cultured until log phase, and then transferred into TAP medium containing 200 mM mannitol for 3 days under the same conditions as the control group. The cell growth rate was calculated using OD values at 750 nm to measure the ability of cells to resist osmotic stress (Additional file 1: Figure S4) [64].

### RNA extraction, de novo assembly, and gene annotation

The E.Z.N.A.® Plant RNA Kit (OMEGA, Germany) was used to extract total RNA from seaweed samples that underwent desiccation and rehydration treatments. High-quality RNA was used for an RNA-sequencing (RNA-seq) analysis, which was completed with an Illumina HiSeq™ 2000 system at the Majorbio BioTech Co. Ltd. (Shanghai, China). The details of gene assembly and annotation were previously provided by Xie et al (2013) [65]. Gene expression levels were calculated based on the RPKM (reads per kb per million reads) method [66].

### Verification by quantitative real-time PCR

First-strand cDNA was synthesized from 1 μg total RNA with the PrimeScript™ RT reagent Kit with gDNA Eraser (TaKaRa, Japan). All cDNA samples were diluted with nuclease-free water to 5 ng μL^− 1^ before being analyzed in a quantitative real-time polymerase chain reaction (qRT-PCR) assay, which was performed in 96-well plates with an ABI 7300 Real-time PCR Detection system. The ubiquitin-conjugating enzyme gene (*PhUBC*) served as a control to normalize target gene expression levels [67]. Details regarding the gene-specific qRT-PCR primers are provided in Additional file 1: Table S1. The qRT-PCR program was as follow: 95 °C for 30 s, 40 cycles of 95 °C for 5 s and 60 °C for 31 s. The qRT-PCR assay was performed in triplicate for each sample.

### Proteomic analysis

Previously described methods were used for extracting, digesting, and labeling proteins [15]. The average value of three biological replicates and three technical replicates was calculated to represent the final protein abundances at each time point. Proteins with a 1.2-fold change in abundance between samples (*p*-value < 0.05) were determined as DEPs.

### Validation of differentially expressed proteins using a multiple reaction monitoring analysis

Samples were extracted and digested as described and then spiked with 50 fmol β-galactosidase to normalize the data. Multiple reaction monitoring (MRM) analyses were completed with a QTRAP 5500 mass spectrometer (SCIEX, Framingham, MA, USA) equipped with LC-20 AD nanoHPLC system (Shimadzu, Kyoto, Japan). The generated raw data file was integrated with Skyline software. All the transitions for each peptide were used for quantification unless interference from the matrix was detected. A sample spiked with β-galactosidase was used for lable-free data normalization. We used MSstats with the linear mixed-effects model, and the *p* values were adjusted to control the false discovery rate at a cutoff of 0.05. All the proteins having at least 1.2-fold changes in abundance (*p <* 0.05) were considered significant.

### Functional classification and enrichment analysis

DEPs were functionally classified according to MapMan ontology [68]. An enrichment analysis was conducted with the Singular Enrichment Analysis tool of the agriGO toolkit [69]. The enriched metabolic pathways associated with the responsive proteins were identified based on the information in the Kyoto Encyclopedia of Genes and Genomes pathway database.

### Isolation of the transketolase gene and vector construction

The complete cDNA of transketolase gene of *P. haitanensis* (*PhTKL*) was cloned using gene-specific primers (Additional file 1: Table S2). The pChlamy_3 vector (Invitrogen, USA) was used to construct the transformation vector. The PCR-amplified *PhTKL* sequence and the pChlamy_3 vector were digested with *Kpn*I and *Xba*I (TaKaRa, Japan), after which they were ligated to generate the pChlamy-PhTKL recombinant plasmid.

### *Chlamydomonas reinhardtii* nuclear transformation

*Chlamydomonas reinhardtii* cells were transformed according to a modified version of an electroporation procedure described by Yamano et al. (2013) [70]. The *C. reinhardtii* cells were cultured until the cell density reached 1~2 × 10^6^ cells mL^− 1^. 10 mL aliquot of cultured cells was collected by centrifugation at 600×g for 5 min and re-suspended in 200 μL TAP medium containing 40 mM sucrose for a final density of 1 × 10^8^ cells mL^− 1^. Then, 2 μg pChlamy-PhTKL plasmid was linearized with KpnI and added to the cell suspension. The cell suspension was placed into an electroporation cuvette (ECM830, USA). Parameters were optimized as three Pps of 300 V with a 6 ms pulse length and a 50 ms pulse interval. After the electroporation, the cell suspension was transferred to 10 mL TAP medium containing 40 mM sucrose and incubated under dim light (1~2 μmol photons m^− 2^ s^− 1^) for 24 h. Transformed colonies were identified after a 3-day growth period on agar-solidified TAP medium containing 10 μg/mL hygromycin. Cell growth was monitored based on the cell counts determined by measuring the optical density at 750 nm [64].

### Statistical analysis

The qRT-PCR analysis of *PhTKL* expression was repeated three times. Data were recorded as the means ± standard deviations. The significance of any differences between the treatment and control values was determined with a one-way ANOVA and the LSD post hoc test in SPSS 12.0 (SPSS Inc., Chicago, IL, USA) (*p* < 0.05).

## Supplementary information

**Additional file 1: Table S1.** Gene-specific qRT-PCR primers for verifying differentially expressed genes in *Pyropia haitanensis.* **Table S2.** Gene-specific primers for cloning *PhTKL* and quantifying its expression. **Table S3.** Summary of the *Pyropia haitanensis* transcriptome. **Table S4.** Important differentially expressed genes in *Pyropia haitanensis* under desiccation and rehydration conditions. **Figure S1.** Relative expression of unigenes in *Pyropia haitanensis* under desiccation conditions as determined by qRT-PCR. **Figure S2.** Comparison of the changes in mRNA levels and protein abundances in *Pyropia haitanensis*. The relative changes are presented on a log2 scale: (A) 30% vs 0%, (B) 60% vs 0%, (C) 90% vs 0%, and (D) rehydration vs 0%. Different colored spots represent the following results: red, mRNA levels and protein abundances changed significantly; green, only the mRNA levels changed significantly; blue, only the protein abundances changed significantly; black, neither the mRNA levels nor protein abundances changed significantly (*p* > 0.05). **Figure S3.** Verification of the presence of *PhTKL* in transgenic *Chlamydomonas reinhardtii.* **Figure S4.** Correlation between *C. reinhardtii* culture OD_750_ and cell number. The OD_750_ values of the four cultures used for the experiment were 0.0962 ± 0.0010, 0.1544 ± 0.0008, 0.2261 ± 0.0049 and 0.3250 ± 0.0016 respectively

## Data Availability

All supporting data are included within the article or in the additional files.

## Abbreviations

2-DE: Two-dimensional electrophoresis
DEGs: Differently expressed genes
DEPs: Differently expressed proteins
PBPs: Phycobiliproteins
PCA: Principal component analysis
*PhUBC*: Ubiquitin conjugating enzyme gene
PSI: Photosystem I
PSII: Photosystem II
qRT-PCR: Quantitative real-time PCR
ROS: Reactive oxygen species
TAP: Tris-acetate-phosphate
TCA: Tricarboxylic acid
TKL: Transketolase
TPS: Trehalose-6-phosphate synthase
WT: Wild-type
