# Supplementary material for: Insight into transketolase of Pyropia haitanensis under desiccation stress based on integrative analysis of omics and transformation
Source: BMC Plant Biol. 2019 Nov 6;19:475. doi: 10.1186/s12870-019-2076-4 (PMC6836531; doi:10.1186/s12870-019-2076-4)
Supplement: Supplementary file 1 — Additional file 1: Table S1. Gene-specific qRT-PCR primers for verifying differentially expressed genes in Pyropia haitanensis. Table S2. Gene-specific primers for cloning PhTKL and quantifying its expression. Table S3. Summary of the Pyropia haitanensis transcriptome. Table S4. Important differentially expressed genes in Pyropia haitanensis under desiccation and rehydration conditions. Figure S1. Relative expression of unigenes in Pyropia haitanensis under desiccation conditions as determined by qRT-PCR. Figure S2. Comparison of the changes in mRNA levels and protein abundances in Pyropia haitanensis. The relative changes are presented on a log2 scale: (A) 30% vs 0%, (B) 60% vs 0%, (C) 90% vs 0%, and (D) rehydration vs 0%. Different colored spots represent the following results: red, mRNA levels and protein abundances changed significantly; green, only the mRNA levels changed significantly; blue, only the protein abundances changed significantly; black, neither the mRNA levels nor protein abundances changed significantly (p > 0.05). Figure S3. Verification of the presence of PhTKL in transgenic Chlamydomonas reinhardtii. Figure S4. Correlation between C. reinhardtii culture OD750 and cell number. The OD750 values of the four cultures used for the experiment were 0.0962 ± 0.0010, 0.1544 ± 0.0008, 0.2261 ± 0.0049 and 0.3250 ± 0.0016 respectively [file 12870_2019_2076_MOESM1_ESM.pdf]

Additional Table 1 Gene-specific qRT-PCR primers for verifying differentially expressed genes in *Pyropia haitanensis*

Gene ID	Primer sequence	Annotation
c8019_g2	QF: CAACCAATCCAAGCGCACCA	Glutamyl-tRNA reductase
	QR: CCGTTGGGTCTGTGACCTGT	
c13721_g2	QF: CGATGGGAGCCTGTGCAAAC	Fructose-1,6-biphosphate aldolase
	QR: ACACTTCCACCGCTTCACCA	
c13247_g5	QF: TACTTTACGGAGCCCATCGC	Ribose-5-phosphate isomerase
	QR: CACAATCACCTCGCTCGTCA	
c13285_g1	QF: CAGCACCATCTTGCCAATCG	Catalase
	QR: GAGGGGTTATGCAAGGCAGT	
c13697_g1	QF: GCCAGGTAGTGGCGGATCTT	Triosephosphate isomerase
	QR: CGACTGGAAGGACGTGGTGA	
c14453_g1	QF: ATCCCGGTCCGTCTTTAGCG	Glucose-6-phosphate isomerase
	QR: TGCCACCGGCAAGGTCATTA	
c14325_g1	QF: GACCGACGACACGGAGATGT	Ferrochelatase
	QR: GCTCTGGAAGGCGATGGAGT	
c13777_g1	QF: GCTGTTCAGTTGGTTCGCTG	Myo-inositol dehydrogenase
	QR: AGATACCGCGCCATGTCAAA	
c13336_g3	QF: CTTCCTCGTCTCCATCGTCG	Na+-ATPase
	QR: CCGCAATCAGCACCTTGTTC	
c12358_g2	QF: TCACGGCACAACAGTCATCA	Chaperonine 60
	QR:	

	CGCACGCTTTACTGCTTGTT	
c13556_g2	QF: CTCCCTTCCACCTCCCTCCT	Carbonic anhydrase
	QR: CGCAAGCTGACGTGCTACAG	
c2591_g1	QF: CGGTCGTAACCGTCGAGTAG	Glycerol-3-phosphate dehydrogenase
	QR: TTCACCACGTCCTCAATGGG	
c11631_g1	QF: CGTGTTGGTCAGCACATCCG	Glyoxylate reductase
	QR: ATGACGCCCTCCTCTGCTTC	
c12816_g1	QF:ACCTGGGGAGTTTGGGTTTG	Light-harvesting protein
	QR: AATGGCAATCATGGCAAGGC	
c12118_g1	QF: GCTTGGCGTGGATGATTACC	Cu-Zn superoxide dismutase
	QR: GGGACCTCGGCAACCTATT	
c13414_g2	QF: CGACACGTTCAGGATACCGT	Heat shock protein 70
	QR: GACGTATGCGGACAACCAGA	
c13719_g1	QF: ACTGCAGGGCGAGGTACAAA	Carbonic anhydrase 1
	QR: GGTTATGAGTGCCGGGTGGA	

Additional Table 2 Gene-specific primers for cloning *PhTKL* and quantifying its expression

Purpose	Primer name	Organism	Sequences
PCR	TKLF	*P. haitanensis*	GGGTACCCTTCGCCGATCTTTG
	TKLR		CTTCTAGATTAGAGGGCCGACG
qRT-PCR	TKLQF	*P. haitanensis*	TGGGCAAGCTGATTGTGATG
	TKLQR		ATCAATGCTCGCAACGTCAA
Housekeeping gene	TubulinQF	*C. reinhardtii*	CTCGCTTCGCTTTGACGGTG
	TubulinQR		CGTGGTACGCCTTCTCGGC

Additional Table 3: Summary of the *Pyropia haitanensis* transcriptome

Item	Number
Total sequence base	25,951,927
Total sequence num	36,343
Q20（%）	96.93
GC percentage（%）	66.15
Largest transcript（bp）	48,488
Smallest transcript（bp）	201
Average length（bp）	714.08
N50 （bp）	1,199

Additional Table 4: Important differentially expressed genes in *Pyropia haitanensis* under desiccation and rehydration conditions

Gene ID	RPKM ratio to control				Gene annotation
	30%	60%	90%	R	
**Photosynthesis**					
c26748_g1	1.05	0.21	0.19	1.15	Carbonic anhydrase [*Pyropia haitanensis*]
c8855_g1	1.74	1.45	1.32	0.29	Phycocyanin beta subunit [*Pyropia haitanensis*]
c13921_g1	2.62	2.22	2.10	1.67	Light-harvesting complex I polypeptide [*Chondrus crispus*]
c12816_g1	6.15	6.74	6.21	3.83	Light-harvesting protein [*Pyropia yezoensis*]
c13855_g4	2.91	2.69	2.65	1.42	Phycobilisome core linker polypeptide [*Chondrus crispus*]
c13884_g1	2.14	1.77	1.89	1.32	Phycobilisome 31.8kD linker polypeptide [*Chondrus crispus*]
c16802_g1	2.72	0.66	0.65	1.15	PsbA [*Pyropia haitanensis*]

c19722_g1	1.12	0.56	0.60	1.12	PsbB [*Pyropia yezoensis*]
c8583_g1	0.93	0.34	0.52	1.08	PsaA [*Pyropia yezoensis*]
c10780_g1	0.56	2.70	2.44	0.60	Ferredoxin [*Pyropia haitanensis*]
c12565_g1	3.35	3.97	3.25	1.65	PsbO [*Chondrus crispus*]
c13900_g1	2.22	2.06	1.99	0.97	PsbQ [*Guillardia theta*]
c1507_g1	2.06	2.19	1.97	1.22	PsbU [*Chondrus crispus*]
**Protein synthesis and degradation**					
c13722_g1	3.14	3.98	3.15	1.35	Glutamine synthetase II [*Bangia atropurpurea*]
c9501_g2	0.48	0.67	0.52	0.83	Translation initiation factor eIF-5 [*Galdieria sulphuraria*]
c20284_g1	0.07	0.49	0.23	0.66	Translation elongation factor eEF3 [*Chondrus crispus*]
c11312_g1	0.44	0.43	0.50	0.97	Eukaryotic translation initiation factor 4A [*Pyropia yezoensis*]
c12947_g1	0.74	0.48	0.58	0.64	Tryptophan synthase beta chain [*Galdieria sulphuraria*]
c11177_g2	0.92	0.42	0.74	0.40	Lysyl-tRNA synthetase [*Chondrus crispus*]
c12579_g1	0.81	2.52	2.49	0.43	Phenylalanyl-tRNA synthetase beta chain [*Pyropia haitanensis*]
c11236_g3	0.46	0.29	0.20	0.30	Ubiquitin-activating enzyme [*Gracilaria lemaneiformis*]
c11402_g1	0.47	0.56	0.56	0.70	Ubiquitin-protein ligase E3 [*Galdieria sulphuraria*]
c6941_g1	0.63	0.27	0.40	0.58	Ubiquitin carrier protein [*Chondrus crispus*]
**Response to stimuli**					
c12118_g1	2.63	1.39	2.02	0.46	Cu-Zn superoxide dismutase [*Pyropia haitanensis*]
c13285_g1	6.45	2.22	1.68	21.91	Catalase [*Pyropia yezoensis*]
c13138_g2	1.44	1.19	1.21	2.10	Hsp90 co-chaperone p23 [*Cyanidioschyzon merolae* strain 10D]

c13184_g1	0.36	0.30	0.35	0.43	DnaJ [*Pyropia yezoensis*]
c13414_g2	0.19	0.09	0.16	0.26	Heat shock protein 70-1 [*Pyropia haitanensis*]
c10371_g2	0.31	0.14	0.25	0.45	Heat shock protein 70-4 [*Pyropia haitanensis*]
c12646_g1	0.40	0.35	0.38	1.12	Heat shock protein 70-5 [*Pyropia haitanensis*]
c12929_g2	0.49	0.37	0.44	1.02	Heat shock protein 90-1 [*Pyropia haitanensis*]
c12601_g1	0.27	0.13	0.24	0.39	Heat shock protein 90-2 [*Pyropia haitanensis*]
c2591_g1	1.13	2.45	1.15	3.94	Glycerol-3-phosphate dehydrogenase [*Pyropia haitanensis*]
c11321_g1	1.86	1.99	2.01	0.91	Trehalose-6-phosphate synthase [*Pyropia haitanensis*]
**Cell wall and cytoskeleton**					
c3997_g2	8.57	12.20	11.40	2.46	Cell wall protein GP2 [*Chlamydomonas reinhardtii*]
c13878_g1	0.72	2.88	6.44	2.27	Vegetative cell wall protein [*Chlamydomonas incerta*]
c10478_g1	3.12	4.71	1.44	1.32	Extensin [*Volvox carteri*]
c13918_g3	2.90	4.61	3.68	2.53	Leucine-rich repeat extensin-like protein 3 [*Prunus mume*]
c14966_g1	1.46	5.01	3.75	3.13	Formin 2A [*Physcomitrella patens*]
c14009_g1	5.62	8.51	8.15	2.71	Formin 2B [*Physcomitrella patens*]
c12752_g6	0.94	6.64	1.33	0.97	Formin-like protein 18 [*Triticum urartu*]
c4318_g1	0.53	2.11	1.21	1.27	Formin-like protein 5 [*Oryza sativa Japonica Group*]
c12205_g1	1.39	5.06	3.66	2.52	Pherophorin-dz1 protein [*Volvox carteri* f. nagariensis]
c13596_g2	4.56	11.94	6.86	14.56	Plus agglutinin [*Chlamydomonas incerta*]
c13502_g2	0.88	3.51	1.55	1.06	UDP-glucose pyrophosphorylase [*Eucheuma denticulatum*]
**Energy and carbohydrate metabolism**					

c13697_g1	2.00	3.98	2.46	1.03	Triosephosphate isomerase [*Pyropia haitanensis*]
c12026_g2	1.47	2.08	1.85	1.43	Glyceraldehyde 3-phosphate dehydrogenase [*Pyropia yezoensis*]
c8681_g1	0.62	2.53	2.01	1.61	Phosphoenolpyruvate carboxykinase [*Galdieria sulphuraria*]
c13882_g2	6.20	42.26	16.85	4.65	Phosphoglycerate kinase [*Pyropia yezoensis*]
c13739_g1	1.92	3.65	2.58	1.47	Enolase [*Chondrus crispus*]
c13721_g1	2.40	6.52	3.83	0.79	Fructose-bisphosphate aldolase [*Pyropia yezoensis*]
c13721_g2	4.40	26.78	11.24	1.86	Fructose-1,6-biphosphate aldolase [*Chondrus crispus*]
c11029_g1	0.84	1.98	1.24	0.49	Phosphoglucomutase [*Chondrus crispus*]
c11550_g2	1.19	1.01	0.94	2.07	Fatty acid beta-oxydation multifunctional protein [*Chondrus crispus*]
c11976_g1	0.93	0.41	0.69	0.40	Pyruvate kinase [*Chondrus crispus*]
c28423_g1	0.88	2.60	1.52	1.13	V-type ATP synthase [*Chondrus crispus*]
c9781_g2	0.64	1.59	0.92	0.17	Pyruvate dehydrogenase E1 component [*Pyropia haitanensis*]
c6939_g2	2.23	6.34	3.59	1.07	Chloroplast fructose-1,6-bisphosphatase [*Pyropia haitanensis*]
c2488_g1	0.93	2.64	1.49	0.49	Inorganic pyrophosphatase [*Chondrus crispus*]

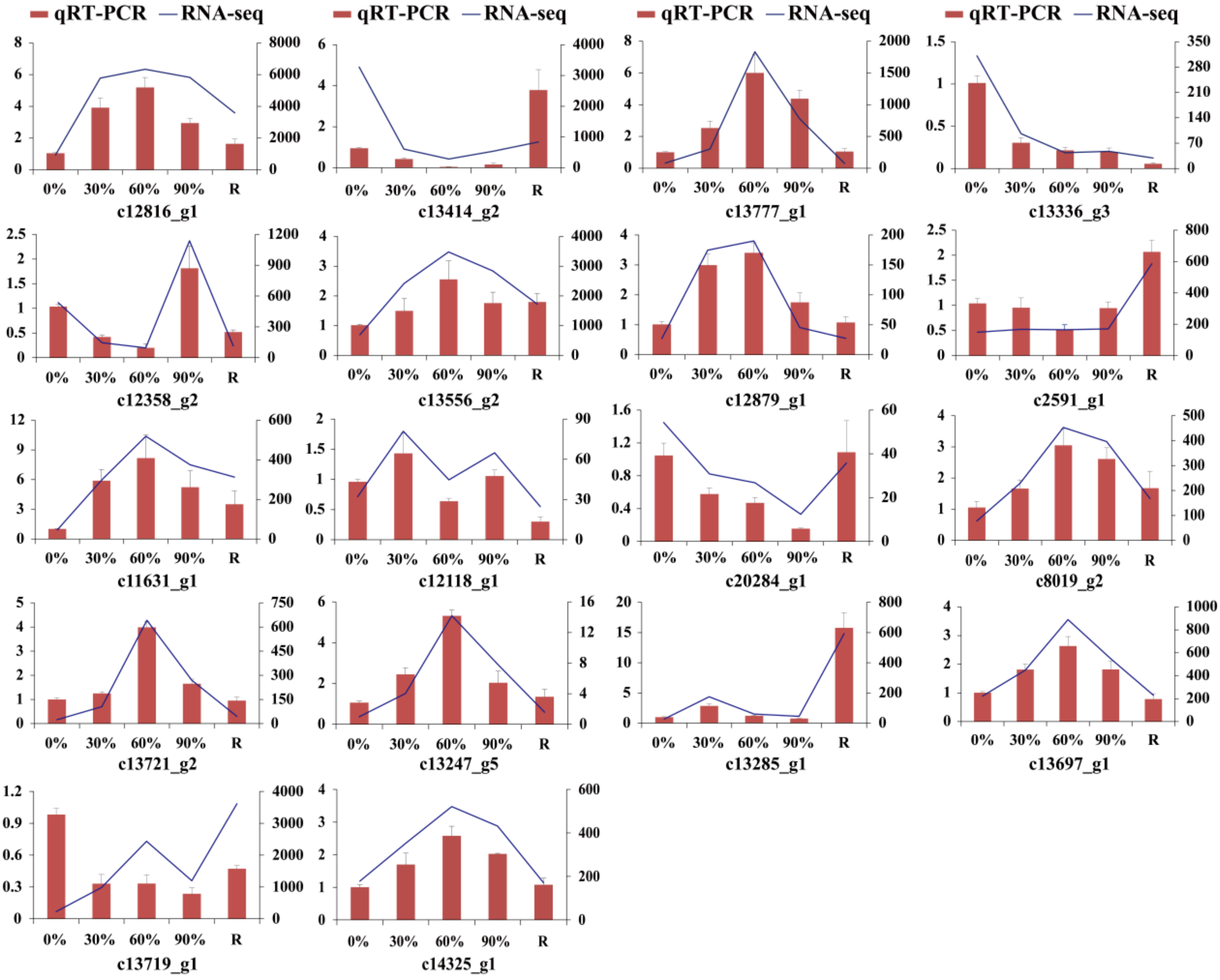

Additional Figure 1: Relative expression of unigenes in *Pyropia haitanensis* under desiccation conditions as determined by qRT-PCR

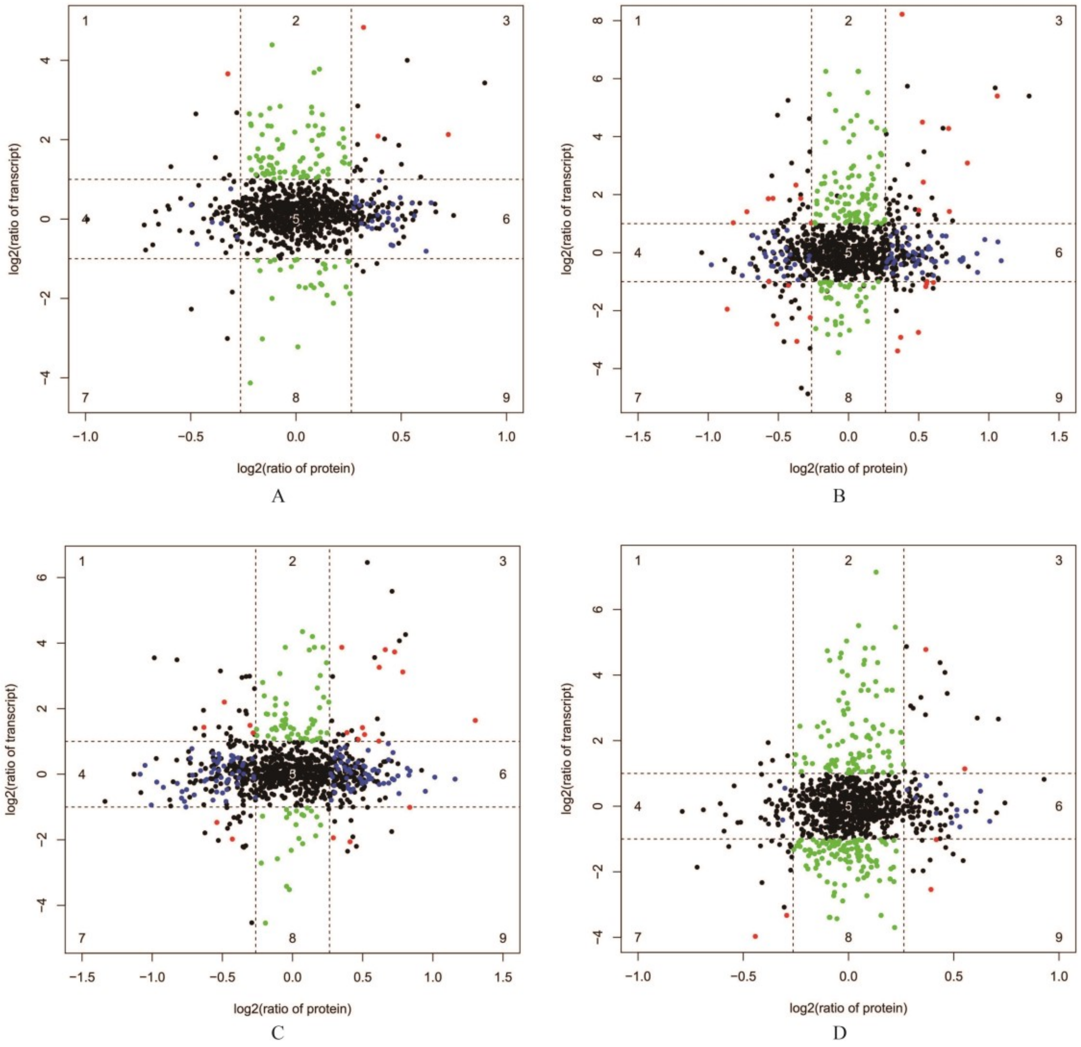

Additional Figure 2: Comparison of the changes in mRNA levels and protein abundances in *Pyropia haitanensis*. The relative changes are presented on a log2 scale: (A) 30% *vs* 0%, (B) 60% *vs* 0%, (C) 90% *vs* 0%, and (D) rehydration *vs* 0%. Different colored spots represent the following results: red, mRNA levels and protein abundances changed significantly; green, only the mRNA levels changed significantly; blue, only the protein abundances changed significantly; black, neither the mRNA levels nor protein abundances changed significantly ($p > 0.05$).

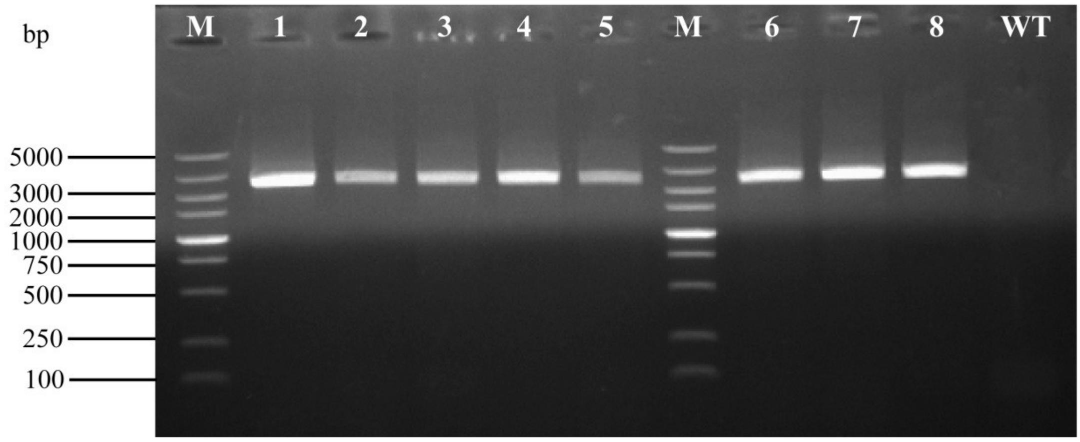

Additional Figure 3 Verification of the presence of *PhTKL* in transgenic *Chlamydomonas reinhardtii*

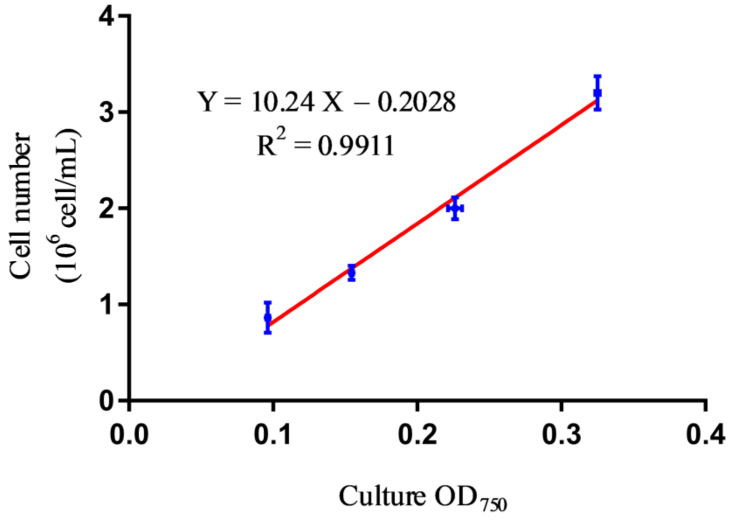

Additional Figure 4 Correlation between *C. reinhardtii* culture OD_{750} and cell number. The OD_{750} values of the four cultures used for the experiment were 0.0962±0.0010, 0.1544±0.0008, 0.2261±0.0049 and 0.3250±0.0016 respectively
